# A novel candidate gene *CLN8* regulates fat deposition in avian

**DOI:** 10.1186/s40104-023-00864-x

**Published:** 2023-05-01

**Authors:** Xiaoqin Li, Fan Zhang, Yunxiao Sun, Dandan Sun, Fangxi Yang, Yongtong Liu, Zhuocheng Hou

**Affiliations:** 1 National Engineering Laboratory for Animal Breeding and Key Laboratory of Animal Genetics and Breeding, College of Animal Science and Technology, MARA, China Agricultural University, No. 2 Yuanmingyuan West Rd, Beijing, 100193 China; 2 Beijing Nankou Duck Breeding Inc, Beijing, 100076 China

**Keywords:** Avian, Adipogenesis, *CLN8*, SNP, Transcription regulation

## Abstract

**Background:**

The fat deposition has a crucial role in animal meat flavor, and fat deposition-related traits are vital for breeding in the commercial duck industry. Avian fat-related traits are typical complex phenotypes, which need a large amount of data to analyze the genetic loci.

**Results:**

In this study, we performed a new phenotypic analysis of fat traits and genotyped whole-genome variations for 1,246 ducks, and combed with previous GWAS data to reach 1,880 ducks for following analysis. The carcass composition traits, subcutaneous fat weight (SFW), subcutaneous fat percentage (SFP), abdominal fat weight (AFW), abdominal fat percentage (AFP) and the body weight of day 42 (BW42) for each duck were collected. We identified a set of new loci that affect the traits related to fat deposition in avian. Among these loci, ceroid-lipofuscinosis, neuronal 8 (*CLN8*) is a novel candidate gene controlling fat deposition. We investigated its novel function and regulation in avian adipogenesis. Five significant SNPs (the most significant SNP, *P*-value = 21.37E−12) and a single haplotype were detected in the upstream of *CLN8* for subcutaneous fat percentage. Subsequently, luciferase assay demonstrated that 5 linked SNPs in the upstream of the *CLN8* gene significantly decreased the transcriptional activity of *CLN8*. Further, ATAC-seq analysis showed that transcription factor binding sites were identified in a region close to the haplotype. A set of luciferase reporter gene vectors that contained different deletion fragments of the *CLN8* promoter were constructed, and the core promoter area of *CLN8* was finally identified in the −1,884/−1,207 bp region of the 5′ flanking sequences, which contains adipogenesis-related transcription factors binding sites. Moreover, the over-expression of *CLN8* can remarkably facilitate adipocyte differentiation in ICPs. Consistent with these, the global transcriptome profiling and functional analysis of the over-expressed *CLN8* in the cell line further revealed that the lipid biosynthetic process during the adipogenesis was significantly enriched.

**Conclusions:**

Our results demonstrated that *CLN8* is a positive regulator of avian adipocyte differentiation. These findings identify a novel function of *CLN8* in adipocyte differentiation, which provides important clues for the further study of the mechanism of avian fat deposition.

**Supplementary Information:**

The online version contains supplementary material available at 10.1186/s40104-023-00864-x.

## Background

Ducks have been widely reared worldwide for their meat, egg, down, and feathers. Pekin duck is a famous breed all over the world for its extensive adaptability, rapid growth, and superior taste owing to the superior adipose deposition [1]. Pekin duck also is an important raw material source for Pekin roast duck which needs high subcutaneous fat content to ensure its fine flavor [2].

Fat deposition has a crucial role in animal meat flavor. The coordinated processes of fat synthesis and deposition were observed in the liver and fat tissue, respectively [3, 4]. Avian has been utilized as a good animal model for exploring basic adipogenesis mechanisms [5]. Adipogenesis involves a temporally regulated set of gene-expression events [6], and a set of studies regarding subcutaneous and abdominal fat have been previously investigated in avian [7–9]. In a genome-wide association study (GWAS) analysis for fat traits across poultry breeds, significant associations were identified on 21 QTLs for skin fat traits and 396 QTLs for abdominal fat traits in a variety of chicken chromosomal regions [10]. Examining currently available meat quality studies, hundreds of QTLs have been curated in pigs, cattle, and chickens [11]. However, owing to a lack of microarrays and cheap genotyping methods in ducks, few studies have been reported using whole genome-sequencing data [12, 13]. Recently, we applied for the first time the genotyping-by-sequencing (GBS) method in ducks [14–16], which makes it possible to detect meat quality-related QTLs. Further, some newly identified candidate genes were discovered for fat-deposition and meat-quality traits, which reported fat-deposition and meat-quality traits in Pekin ducks [17]. Avian fat-related traits are typical complex phenotypes, which need a large amount of data to analyze the genetic loci. In this study, we collected large-scale duck fat traits and targeted genome-wide variation using resequencing methods, aiming to identify loci with pleiotropy for subcutaneous and abdominal fat traits by GWAS.

In this study, we first phenotyped fat traits and genotyped whole-genome variations for 1,246 ducks, combined with the previous GWAS data, we obtained a group of new loci that affect the traits related to avian fat deposition. Among them, *CLN8* is a new gene with great potential in regulating fat deposition. The functional basis of the most intriguing significant loci was elucidated in the present study. The significant signal was discovered to be driven by 5 SNPs, which were the variants located in 1,920 bp upstream of the *CLN8* gene that alters the binding affinity of several transcript factors and may lead to differential *CLN8* gene expression. Further, this study analyzed the upstream regulatory region of *CLN8* by a dual-luciferase reporter system and found that the important haplotype mutation of the regulatory region composed of 5 SNPs obtained by GWAS analysis, which lead to the decreased transcriptional activity of *CLN8*. In further, we performed gain-of-function experiments on the candidate gene *CLN8*, showing that *CLN8* is a promoter of adipocyte differentiation. Our findings provide a theoretical basis for the functional analysis and regulatory mechanism of *CLN8* in avian adipogenesis and propose a novel function of *CLN8* in avian adipocyte differentiation.

## Materials and methods

### Phenotype collection

Two batches of ducks were reared and phenotypes were collected. This study reared about 1,400 ducks from the day-old ducklings which are inherited from randomly mated Pekin ducks. Each batch included about 700 ducks and was reared in a large population for 21 d, and then all ducks were recorded feed consumption individually to 42 d. All ducks were fed the same diet and maintained the same lighting conditions as described previously [18]. At 41 d, blood was collected from the brachial vein of the duck by venipuncture using citrated syringes during a routine health inspection. Ducks were weighed and killed by stunning and exsanguination, after withholding feeding for 6 h at 42 d. After the carcass composition traits were determined, meat quality traits were measured using methods previously described in detail [17], including subcutaneous fat weight (SFW), subcutaneous fat percentage (SFP), abdominal fat weight (AFW), abdominal fat percentage (AFP) and the body weight of 42 d (BW42). Finally, this study collected 1,246 ducks with full phenotypes for further whole-genome genotyping. We also combined with previously published data, and finally, a total of 1,880 (males, 932; females, 948) Pekin ducks were used in this study.

### DNA extraction and whole genome sequencing

Populations from different years were genotyped using two types of genotyping strategies. Genomics DNA from Population 2014 (Pop2014) with 634 individuals was extracted from blood using the standard phenol/chloroform method, and genotyping by sequencing (GBS) was used for genotyping as described previously [14]. Genomic DNA from Population 2019 (Pop2019) and Population 2020 (Pop2020) with total of 1,246 ducks was extracted using the Qiagen kit (QIAGEN, Valencia, CA, USA), and samples were subjected to next-generation sequencing (NGS) using Illumina paired-end cycles. The whole genome sequencing of all 1,880 duck samples was available publicly. The data were deposited in the NCBI sequence read archive (PRJNA506902, SRP068685 and PRJNA921894).

### Variant calling and annotation

Adaptors and low-quality sequences were removed using the Picard software (http://picard.sourceforge.net/) (version 2.28). High-quality reads were aligned to the Mallard reference genome (ASM874695v1, CAU_wild_1.0) using the BWA-MEM algorithm of the BWA software package (v0.7.17) [19]. Duplicate reads were excluded using the Picard tool MarkDuplicates. Variant calling was performed with the GATK HaplotypeCaller (v4.1.8) and using joint genotyping across all sequenced samples [20]. PLINK (v1.922) was used to exclude SNPs and individuals with more than 5% missing data, markers with minor allele frequency < 1%, and sex chromosome related SNPs. Variant data were imputed using Beagle (v5.1) [21]. After applying these filters, 8,448,069 SNPs and 1,880 individuals were retained for further analysis. Known variants from the Ensemble variation database (release 10) were used for variant annotation with VEP (v88.9) software [22].

### Statistical genetic analyses and GWAS analysis

The correlation between traits and covariates was examined by calculating Pearson's correlation coefficients. We used Univariate Linear Mixed Models from GEMMA (v0.98.3) to perform association analysis with the Wald test [23, 24], an additive genetic model incorporating birthday, sex, batch, and 5 genetic principal components (PCs) as covariates. PCs were obtained from an LD-pruned dataset of 573,457 SNPs. The number of PCs to be included in the regression was determined by inspecting the proportion of variance explained and by checking PC scatter plots.

### ATAC-seq and analysis of *CLN8* promoter binding region

Fifty thousand nuclei from Pekin duck subcutaneous preadipocytes (*n* = 2) before and after oleic acid-induced preadipocyte differentiation were transposed using Tn5 transposase as previously described [25]. The PE150 ATAC-seq reads were mapped to the Mallard reference genome (GenBank: ASM874695v1) using Burrows-Wheeler Aligner (BWA) with default parameters [19]. SAM files were converted to the BAM format using Samtools (v0.1.19) [26]. The peaks were called using MACS2 in each sample and using the filtered BAM files with the parameters (-g 9.6e8 -q 0.01 -B –SPMR –nomodel -shift -100 -extsize 200) [27]. All the alignment files were scaled to RPKM-normalized read coverage files using DeepTools [28]. The library size factors were estimated to compare binding profiles between different samples in an unbiased manner by the DESeq2 [29]. Differentially accessible regions were detected using DESeq2 with a fold change of more than 1.5 and adjusted *P* value (*Q*-value) below 0.05. The HOMER tool (http://homer.salk.edu/homer/motif/) was used to detect the motifs.

### Plasmid construction

*CLN8* knock-in plasmids: In order to construct the *CLN8*-over-expression vector, the full-length coding sequence of ch*CLN8* (NCBI reference sequence: NM_204214.2) was amplified from chicken subcutaneous adipose cDNA by PCR using the primers listed in Additional file 1, and cloned into the CMV promoter-driven piggyBac and an EF1α promoter-driven cherry plasmid by replacing cherry using NheI and AccI (New England Biolabs, Ipswich, MA, USA). pGL3.1 dual-luciferase reporters: Different length fragments of the upstream area of *CLN8* (NCBI reference sequence: NC_052534.1) were amplified by PCR using the primers listed in Additional file 1 from Pekin duck subcutaneous adipose DNA, and then, cloned into pGL3.1-basic vector. The important candidate mutant fragments were synthesized by Tsingke Biotechnology Co., Ltd., and cloned into pGL3.1-promoter vector.

Gene over-expression vector: The *C/EBP*α over-expression vector was constructed according to the user manual of Easy Ligation Kit (Sidansai, Shanghai, China). *C/EBPα* coding sequence (NCBI reference sequence: NM_001031459.2) was amplified from chicken subcutaneous adipose cDNA by PCR. The PCR product was cloned into the pcDNA3.1 vector. The successful *C/EBP*α over-expression vector, was confirmed by DNA sequencing.

### Cell culture and transfections

A cell line of immortalized chicken preadipocytes (ICPs) [30] was cultured in DMEM/F12 (Gibco, Gaithersburg, MD, USA) supplemented with 10% fetal bovine serum (Hyclone, Logan, UT, USA), and 0.1% penicillin/streptomycin (Invitrogen, Carlsbad, CA, USA). To induce ICPs differentiation, we added 160 µmol/L sodium oleate (Sigma Life Science, St. Louis, MO, USA) to the medium [31].

The pGL3.1 dual-luciferase reporters' transfections were performed with Lipofectamine 2000 reagent (Invitrogen, Carlsbad, CA, USA) according to the manufacturer’s direction. Nucleic acids were diluted in OPTI-MEM Medium (Gibco, Gaithersburg, MD, USA). All experiments were carried out at least three times independently.

For *CLN8*^OE^ cell selection, ICPs were seeded in 6-well plates for further transfection using FuGENE® HD Transfection Reagent (Promega, Madison, WI, USA). After a 48-h recovery period, the cells were supplemented with 1.5 μg/mL of puromycin (Sigma-Aldrich, MO, USA) in the culture medium for 3 d until wild cells dead. Cells were harvested using 0.25% trypsin/EDTA (Gibco, Gaithersburg, MD, USA).

### Oil red O staining and quantification

The cells were washed with PBS and fixed in 4% formaldehyde for 20 min. Then the cells were stained with Modified Oil red O Staining Kit (Beyotime, Jiangsu, China) according to the manufacturer’s manual. Morphological changes were observed and photographed under an inverted fluorescent microscope (Nikon). The Oil red O dyes were then extracted in an isopropanol solution containing 4% Nonidet P-40 and quantified by a Model 680 Microplate Reader (Bio-Rad) at 510 nm.

### Dual-luciferase reporter assay

For the promoter activity assays, ICPs were transfected with the luciferase reporter gene vector which contained different deletion fragments of the *CLN8* promoter or control vector, and the TK-Renilla reporter was also co-transfected to each sample as an internal control using the Lipofectamine 2000 reagent (Invitrogen, Carlsbad, CA, USA) in 24-well plates. After 48 h transfection, cells were washed by PBS twice, and the activities of Firefly and Renilla luciferase were measured according to the manual of Dual-Luciferase Reporter Assay Kit (Vazyme, Nanjing, China), and the relative fluorescence intensity was detected by a microplate reader. All the data were acquired by averaging the results from three independent repeats.

### RNA extraction, cDNA synthesis, and quantitative real-time PCR

Total RNA was isolated from the cells using RNAiso reagent (Takara, Otsu, Japan) according to the manufacturer’s instructions. According to the manufacturer's manual, the reverse transcription reaction for mRNA was performed with the PrimeScript RT reagent Kit (Perfect Real-Time; Takara, Otsu, Japan). Using total RNA as a template and oligo (DT) primer, the first strand of cDNA was inverted. The specific RT-qPCR primers of mRNA were designed using Primer 3 software (version 0.4.0, Howard Hughes Medical Institute). Primer sets are listed in Additional file 1. The amplification efficiency and specificity of all the primers were tested before the formal quantitative experiment. Real-Time fluorescence quantification PCR (RT-qPCR) was performed using TB green premix Ex Taq™ fluorescence quantitative kit (Takara, Otsu, Japan), and the qPCR program was carried out in an ABI-7500 PCR machine (Applied Biosystems, MA, USA). The housekeeping gene GAPDH was used as the internal control, and the method of 2^-ΔΔCt^ was used to quantification the results as described [32]. All reactions were run in triplicate.

### Western blot

Cultured cells were washed with PBS and homogenized with RIPA buffer (Beyotime, Jiangsu, China) containing a protease inhibitor cocktail (Beyotime, Jiangsu, China). Protein concentrations were determined using the BCA Protein Assay Kit (Beyotime, Jiangsu, China). Proteins were denatured and subjected to 12% polyacrylamide gel and transferred to methanol‐activated PVDF membranes. Blots were probed using the primary antibodies: Rabbit polyclonal anti-FLAG antibody for Western blots (1:500; Sigma, Cat. No./F7425), Rabbit polyclonal anti-beta actin antibody, (1:3,000; Proteintech, Cat. No./20536–1-AP), overnight at 4 °C. After 1 h incubation with HRP-conjugated Affinipure goat anti-rabbit IgG (H + L) as a secondary antibody (1:5,000, Proteintech, Cat. No./SA00001-2), immunodetection was performed using enhanced chemiluminescence (ECL) Western blotting substrate (Beyotime, Jiangsu, China) and detected with FluoChem R imaging system (ProteinSimple, CA, USA).

### RNA-Seq analysis

Raw reads were trimmed to remove adapters and low-quality reads, with Trimmomatic (version 0.39) [33]. Read counts for each gene were calculated using Salmon (v1.8.0) [34] and GRCg7b (GalGal1.mat.broiler.GRCg7b) as the reference genome. We used the DESeq2 (v.1.28.1) package [29] to identify differentially expressed genes (DEGs) between *CLN8*^NC^, and *CLN8*^OE^ cells at different days (d 0 and 3). Therefore, samples were excluded from further analysis due to its low global Pearson correlation with the other repeat samples (*R*^2^ < 0.95). Genes with |log_2_FC| ≥ 2 and the Benjamini & Hochberg (BH) adjusted* P*-value (adjusted-*P* value) < 0.05 were considered as DEGs. Transcription factor prediction using an online website (http://gene-regulation.com/pub/programs/alibaba2/index.html) [35].

### Functional annotation and enrichment

Genes were annotated with gene Symbols from the UniProt database for functional annotation. Gene Ontology (GO) and Kyoto Encyclopedia of Genes and Genomes (KEGG) analysis of the enriched genes was performed using the web-based Metascape (http://metascape.org/gp/index.html#/main) [36].

## Results

### Summary of the phenotypes and genotypes

The basic statistics of 42-d body weight, subcutaneous fat weight, subcutaneous fat percentage, abdominal fat weight, and abdominal fat percentage for each population were shown in Additional file 2. The heritability, genetic correlations, and phenotypic correlations of each trait were shown in Table 1. The estimates of heritability range from 0.42 to 0.61. Trait SFW presented the highest heritability (0.61) estimates, while the AFP was the lowest (0.42). The highest phenotypic correlation (0.95) and genetic correlation (0.98) were shown between AFW and AFP, and the lowest phenotypic correlation (0.33) and genetic correlation (0.26) were calculated between BW42 and AFP. The phenotypic and genetic correlations among these fat-related traits (SFW, SFP, AFW, and AFP) were all greater than or equal to 0.62.

**Table 1:** Genetic parameters*

	**BW42**	**SFW**	**SFP**	**AFW**	**AFP**
BW42, g	**0.43**	0.79	0.44	0.57	0.33
SFW, g	0.87	**0.61**	0.84	0.80	0.62
SFP, %	0.50	0.94	**0.49**	0.76	0.71
AFW, g	0.67	0.88	0.87	**0.49**	0.95
AFP, %	0.26	0.70	0.84	0.98	**0.42**

^*^Heritability (bold, diagonal), genetic correlations (below diagonal), and phenotypic correlations (above diagonal) for body weight and fat traits

Abbreviations: *BW42* Body weight at d 42, *SFW* Skin fat weight, *SFP* Skin fat percentage, *AFW* Abdominal fat weight, *AFP* Abdominal fat percentage

The population of Pop2014, Pop2019, and Pop2020 were obtained 1,778 Gb, 4,799.83 Gb, and 4,709.25 Gb clean data, respectively. The average reads mapping alignment rate was 96.57%. The variant calling pipeline identified 14,220,859 SNPs within the duck genomes. After quality filtration and exclusion of variants of uncharacterized chromosomes, 8,448,069 SNPs and 573,457 independent SNPs passed our filters. A principal component analysis (PCA) was constructed to examine the relatedness among three populations (Additional file 3). The PCs showed that all samples were divided into two subpopulations, which were Pop2014 and the other two.

### Genome-wide association study identified the candidate variants and genes

**Fig. 1:**
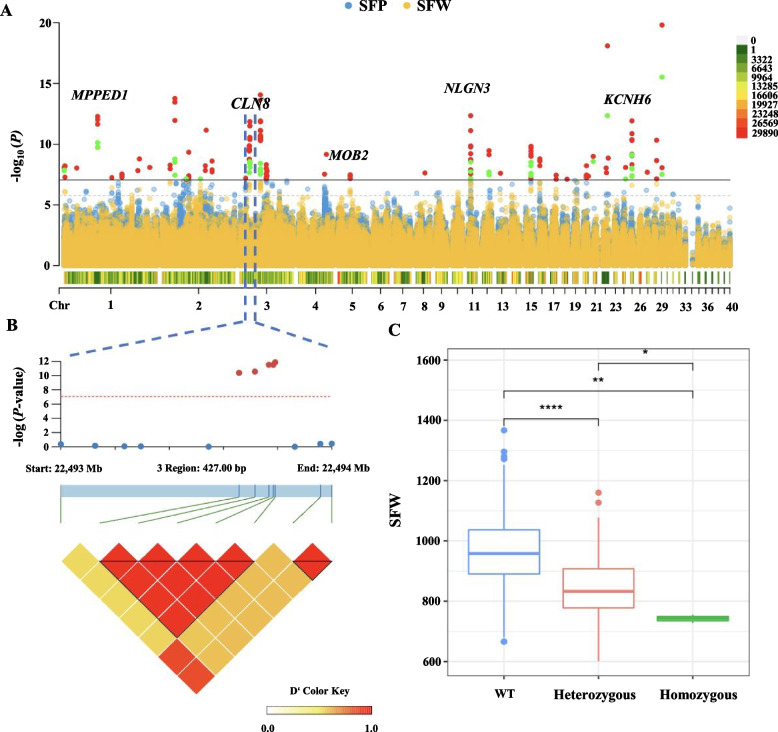
Identification of positional candidate genes for the skin fat phenotype in ducks.** A** Manhattan plot of association results from genome-wide association analysis. *Y* axis shows −log_10_ (*P*-value) of the association result for each SNP. Each SNP is indicated by a colored dot. SNPs are plotted based on the reference duck genome chromosome (*x*-axis). The horizontal solid line is the threshold for the Bonferroni level of significance (*P* < 8.72E−08). The horizontal dashed line is the threshold for the Bonferroni level of significance (*P* < 1.74E−06). **B** Haplotype block at linkage disequilibrium (LD) with the skin fat based on 5 candidate variations. The diagram above the haplotype block indicates genes around candidate variations. **C** The skin fat weight (SFW) of the ducks with different Genotypes. The WT represents the genotype with identical alleles (-T-C-A-A-C) at corresponding chromosomal loci, the Homozygous represents the genotype with identical alleles (-A-T-C–C-G-) at corresponding chromosomal loci, and the Heterozygous represents the genotype with two different alleles (-T-C-A-A-C and -A-T-C–C-G-) at the given locus. The level of significance was presented as ^*^P < 0.05, ^**^P < 0.01, ^***^P < 0.001, ^****^P < 0.0001

A total of 648 chromosome-wide significant SNPs (1/573457, *P* < 1.74E-06) and 242 genome-wide significant SNPs (0.05/573457, *P* < 8.72E-08) across 40 chromosomes were identified by loci-based analysis. We defined candidate genes as genes surrounding significant SNPs in 5 kb extend region. A list of the genome-level significantly associated variants is shown in Additional file 4 and a list of the chromosome-level significantly associated variants is shown in Additional file 5. There were 71, 47, 155, 64, and 14 genome-wide significant SNPs identified for traits BW42, SFW, SFP, AFW, and AFP, respectively. In total, 39 candidate genes were annotated from 68 genome-level significant SNPs. The estimated genomic inflation factor λ ranged from 1.027 to 1.066 among 5 traits (Additional file 6), suggesting no population stratification in the studied population after specifying sex, age, and batch effects as covariates. Manhattan and Q-Q plot of association results from genome-wide association analysis of each trait are shown in Additional file 6. We identified a 7-Mb region between nucleotide position 54 Mb and 61 Mb in chromosome 4 that showed a significant association with the 42-day body weight phenotype with the most significant of 3.86E−10 (rs458603786, T > C, intergenic variant, *P* = 3.86E−10). As for fat traits, two genome-wide significant SNPs (rs117362452, G > A, synonymous variant, and rs291299411, G > C, intron variant) were detected to be correlated with SFW, SFP, AFW, and AFP, which were located at 7.36 Mb on Chr11 and 1.29 Mb on Chr 29. There was a high genetic correlation between subcutaneous fat and abdominal fat, 11 genes (Additional file 5) were common genes between subcutaneous and abdominal fat were identified among a total of 35 significant genes from SFW, SFP, AFW, and AFP. By querying the functional annotations of these genes, we found that *CLN8* is a potential gene affecting lipid synthesis and transport, which was related to SFW, SFP, and AFW. The joint Manhattan plot of SFW and SFP showed in Fig. 1A.

### ATAC-seq analysis of mutations in the upstream of *CLN8*

**Fig. 2:**
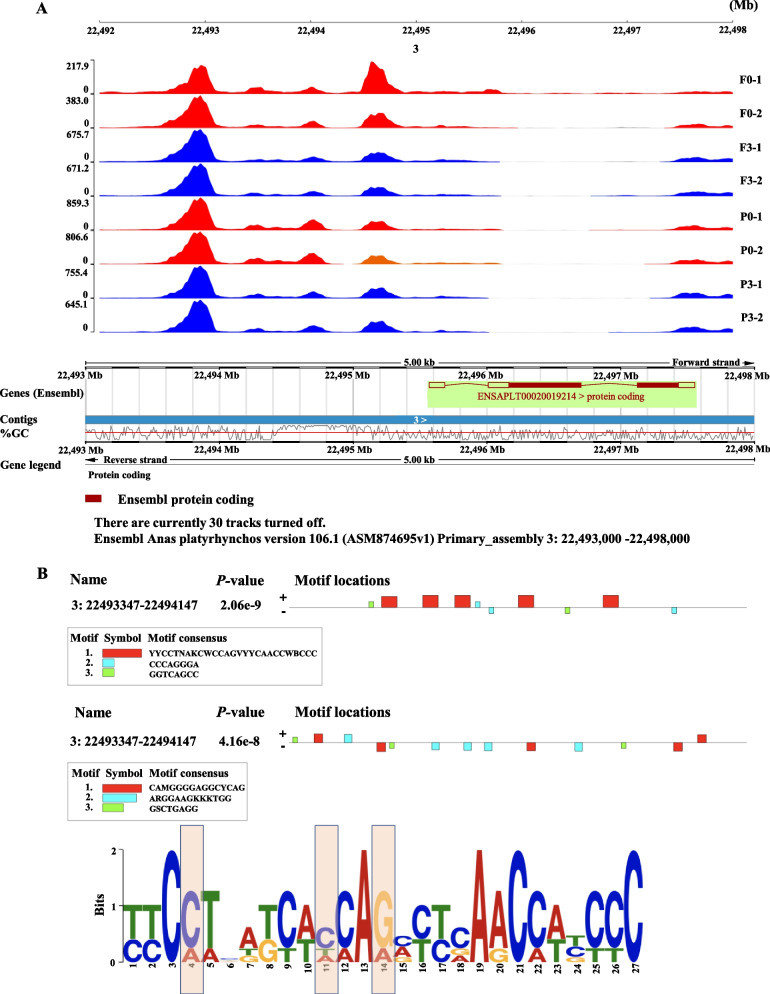
ATAC-seq predicts transcription factor binding sites in the upstream of *CLN8.* **A** Gene structure and nucleosome-free regions fragments in the upstream of *CLN8.* It showed the levels of transposase-accessible chromatin in the region from 22.492 Mb to 22.493 Mb. The P0 and P3 represent subcutaneous preadipocytes (*n* = 2) before (d 0) and after (d 3) oleic acid-induced differentiation respectively. The F0 and F3 represent abdominal preadipocytes (*n* = 2) before (d 0) and F3 after (d 3) oleic acid-induced differentiation, respectively. **B** The Motif binding site in the annotated promoter region. Motif predicted in wild type sequence (up, -T-C-A-A-C-) and in mutant type (down, -A-T-C–C-G-). **C** Sequence logo map of the promoter region, the box area indicated the position of the mutation

The five SNPs (rs322493594, rs322493619, rs322493641, rs322493648, rs322493651) were located in 1,920 bp upstream of the *CLN8* gene on chromosome 3 (Additional file 7). The rs322493651 was the most significant SNP accounting for 1.46% of the genetic variance, and 11.18% of the phenotypic variance. Linkage analysis revealed that they were in high linkage disequilibrium (LD, *r*^2^ > 0.9; Fig. 1B). These results prompted us to speculate that the causative SNPs should be the 5 variations. To confirm the association of the 5 SNPs with the subcutaneous fat phenotype, 616 ducks were genotyped for the *CLN8* heterozygous mutation and 3 for the homozygous, the wild type, and heterozygous mutation of haplotype with 5 SNPs significantly decreased the subcutaneous fat weight (*P* < 0.0001) (Fig. 1C). We used the ATAC-seq to investigate the chromatin accessibility landscape of subcutaneous preadipocytes. Fragments from the nucleosome-free regions (NFR) are expected to be enriched around the transcription start site (TSS) of genes (Additional file 8). Transcription factor binding sites were identified from 22,493,347 bp to 22,494,147 bp, which is located in the upstream of gene *CLN8* (Fig. 2A). To exploit motif information, the JASPAR database was used to predict the motif binding site [37]. Different motif binding site was predicted as shown in Fig. 2B and C between wild-type sequence (up) and mutation sequence (down). ATAC-seq analysis showed that transcription factor binding sites were identified in a region close to the haplotype.

### Active region screening of the *CLN8* promoter

**Fig. 3:**
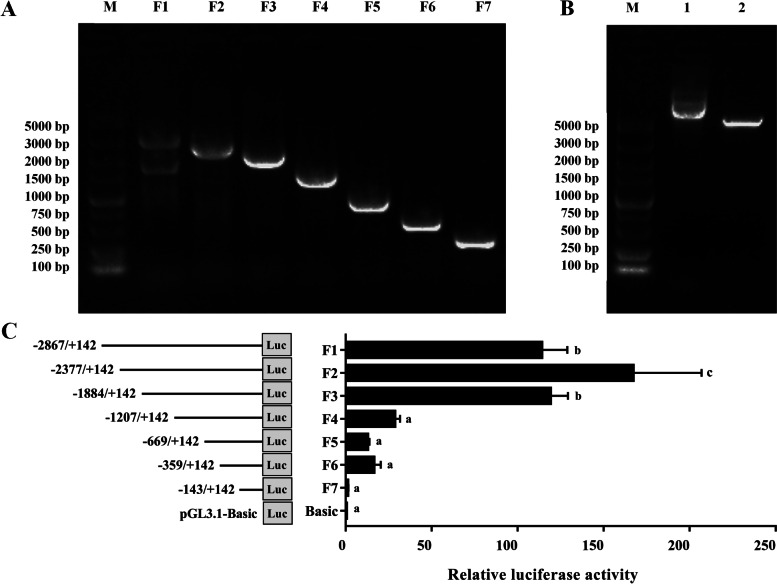
Active region screening of the *CLN8* promoter.** A** Different lengths fragment of *CLN8* promoter cloned by PCR. **B** Plasmid digested of pGL3.1-Basic Vector by NheI and XhoI. **C** Relative luciferase activity of different promoter fragments. F1-F7: Fluorescence activity values detected after transfection of ICP1 cells with fluorescence vectors containing promoter truncated fragments of different lengths, using the pRL-TK as reference. Data are shown as mean ± SD of three biological replicates. An independent sample *t*-test was used to analyze the statistical differences between groups. ^a^^–^^c^Different lowercase letters showed significant differences (*P* < 0.05) in relative luciferase activity between groups

Based on Pekin duck genomic DNA, we designed primers in the proposed promoter region of *CLN8* and amplified seven deletion fragments of different lengths: 3,009 bp, 2,519 bp, 2,026 bp, 1,349 bp, 811 bp, 501 bp, and 285 bp (Fig. 3A). To explore the active region of the *CLN8* promoter, seven truncated fragments of the *CLN8* promoter were amplified and inserted into the pGL3.1-Basic vector (Promega, USA) using restriction enzymes Kpn I and Xho I (Fig. 3B), and were co-transfected into ICP1 cells with the pRL-TK vector for luciferase activity detection. The results showed that the luciferase activity of pGL3.1-F3, pGL3.1-F2, and pGL3.1-F1 were significantly increased compared to that of pGL3.1-Basic, which was the control group (*P* < 0.05), the promoter activity of pGL3.1-F4 was significantly reduced (*P* < 0.05), and the activity of pGL3.1-F3 was significantly increased compared to that of pGL3.1-F4 (*P* < 0.05) (Fig. 3C). These results demonstrated that the region from −1,884 to −1,207 bp upstream of the transcription start site of *CLN8* was the promoter core transcription active region.

### The functional polymorphism SNPs decreased the transcriptional activity of *CLN8*

**Fig. 4:**
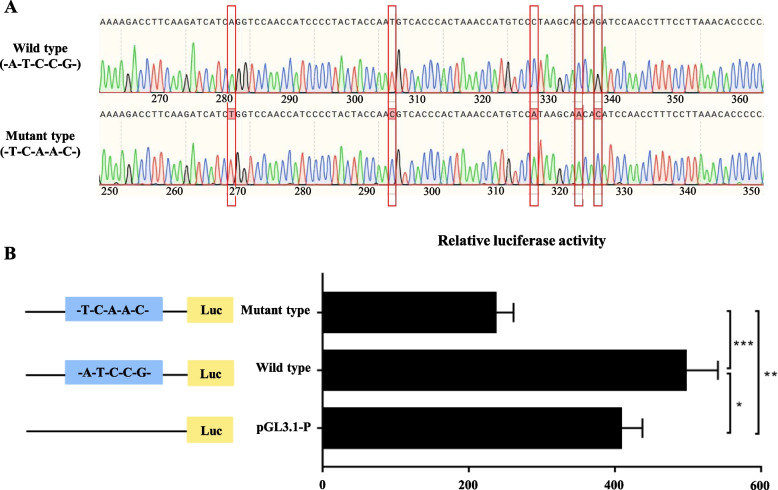
The functional polymorphism SNPs decreased the transcriptional activity of *CLN8*. **A** Wild-type and mutant-type *CLN8* plasmids were verified by direct sequencing. **B** Three luciferase reporter gene constructs were generated. They share identical backbone sequences except for the polymorphisms as left. Significantly lower luciferase activity of the mutant type (-A-T-C–C-G-) haplotype was observed when compared with the wild type (-T-C-A-A-C-) haplotype vectors in the ICP1 cell line. Data are shown as mean ± SD of three biological replicates. An independent sample *t*-test was used to analyze the statistical differences between groups. The level of significance was presented as ^*^P < 0.05, ^*^^*^P < 0.01, ^*^^*^^*^P < 0.001

We next investigated the effect of the 5 SNPs (wild-type: -A-T-C–C-G- and Mutant-type: -T-C-A-A-C-) variant on the transcriptional activity of the *CLN8* gene. Wild and Mutant haplotype *CLN8* plasmids were verified before transfection by direct sequencing (Fig. 4A). To assess the effects of the polymorphisms, we generated two luciferase reporter gene constructs that share identical backbone sequences except for the polymorphisms, as shown in Fig. 4B, reporter gene expression driven by the A-T-C–C-G-containing *CLN8* promoter (wild-type) was greater than that driven by the T-C-A-A-C-containing counterpart (mutant-type) (*P* < 0.001). This result demonstrated that significantly lower transcriptional activity of the mutant haplotype was observed when compared with the wild types.

### *CLN8* promotes the differentiation of avian adipocytes

**Fig. 5:**
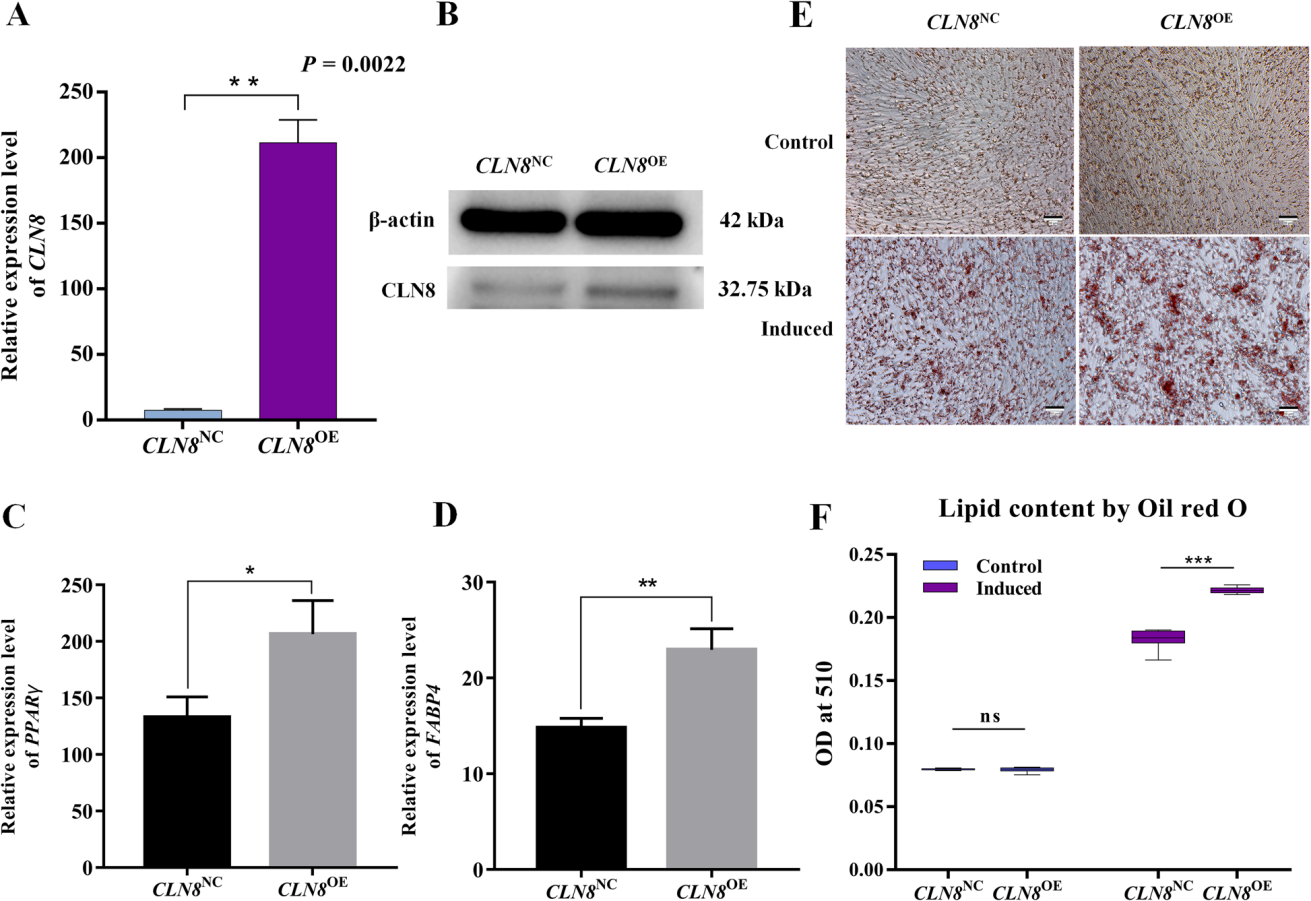
Over-expression with *CLN8* promotes the differentiation of adipocytes. **A** and **B** *CLN8*^OE^ cell significantly promoted CLN8 mRNA and protein expression in ICPs. **C** and **D** mRNA levels of adipocyte genes *PPARγ* and *FABP4* were analyzed with RT-PCR. **E** Representative images of *CLN8*^OE^ cells increased the lipid droplet formation by Oil red O staining on d 3. **F** Comparison of the lipid droplet content of *CLN8*^OE^ and *CLN8*^NC^ cells obtained by Oil red O staining and extraction methods. Data are shown as mean ± SD of three biological replicates. An independent sample *t*-test was used to analyze the statistical differences between groups. The level of significance was presented as ^*^P < 0.05,  ^*^^*^P < 0.01, ^*^^*^^*^P < 0.001

To further determine the roles of *CLN8* in avian adipocyte differentiation, we first performed gain-of-function experiments by using piggyback delivery of *CLN8* into ICPs, and we detected its expression in the overexpression group and control group cells. As shown in Fig. 5A and B, a transfected *CLN8* clone can significantly increase *CLN8* expression at the level of transcription and translation by qPCR and western blot. Further, we detected the expression of adipocyte markers, *PPARγ*, and *FABP4*, and the results showed that they were significantly increased in *CLN8*^OE^ cells (Fig. 5C and D). As shown by Oil red O staining of neutral lipids and detection of the relative lipid content, over-expression of *CLN8* in these cells (*CLN8*^OE^) significantly facilitated lipid formation (Fig. 5E and F). These results indicate that *CLN8* is a positive regulator for the avian adipogenesis.

### *CLN8* is involved in the gene regulation network of preadipocyte differentiation

To better understand the effect of *CLN8* on adipogenesis, we performed mRNA-Seq experiments in *CLN8*^OE^ and *CLN8*^NC^ cells prior to differentiation (d 0) and d 3 after differentiation (Additional file 9, 10). To confirm the results from mRNA-Seq, RT-PCR was performed in the present study firstly. Our RT-PCR results were highly consistent with the mRNA-Seq results (Additional files 11, 12, 13). In total, 2,488 and 2,159 DEGs were up-regulated and down-regulated in *CLN8*^OE^ cells compared to *CLN8*^NC^ cells on d 3, respectively (Additional file 14). GO analysis for DEGs up-regulated in *CLN8*^OE^ cells are enriched for cell differentiation processes, while DEGs up-regulated in *CLN8*^NC^ cells are enriched for the terms related to cell cycle (Table 2; Additional file 15). These results indicate that the knock-in of *CLN8* alone has initiated the preadipocyte differentiation early. Notably, we focused on the DEGs enriched in entries directly related to fat cell differentiation in *CLN8*^OE^ cells compared to the *CLN8*^NC^ group. There are 9 up-regulated DEGs were enriched in the positive regulation of fat cell differentiation (*PTGS2*, *PPARG*, *MAPK14*, *ASXL2*, *PPARD*, *STK4*, *KLF5*, *BMP2*, *ZC3H12A*), and 9 down-regulated DEGs were enriched in the negative regulation of fat cell differentiation (*ARNTL*, *RUNX1T1*, *GATA2*, *CCN4*, *YAP1*, *ANKRD26*, *ZFPM1*, *BBS12*, *ASXL1*) (Additional file 15). These data suggest that *CLN8* is involved in the gene regulation network of avian preadipocyte differentiation.

**Table 2:** The representative GO enrichment analysis terms of DEGs in *CLN8*^OE^ and *CLN8*^NC^ cells

Group	Up-regulated	Gene count	log(*Q*-value)	Down-regulated	Gene count	log(*Q*-value)
*CLN8*^OE^ vs. *CLN8*^NC^ (Day 0)	Regulation of cell growth	49	−11	mRNA metabolic process	98	−17
	Regulation of cell development	52	−9.8	Regulation of cell cycle process	105	−15
	Positive regulation of cellular component biogenesis	46	−7.4	Mitotic cell cycle	89	−14
	Lipid biosynthetic process	43	−4.6	DNA metabolic process	102	−13
	Positive regulation of cell morphogenesis involved in differentiation	11	−2.7	Positive regulation of cell cycle	53	−7.8
*CLN8*^OE^ vs. *CLN8*^NC^ (Day 3)	Positive regulation of cell migration	94	−19	Skeletal system development	72	−13
	Regulation of cell adhesion	107	−16	DNA metabolic process	83	−9.4
	Positive regulation of MAPK cascade	70	−11	Nuclear division	46	−8.7
	White fat cell differentiation	5	−1.9	Mitotic cell cycle	68	−8.2
	Positive regulation of fat cell differentiation	9	−1.2	Negative regulation of fat cell differentiation	9	−1.5

## Discussion

Numerous studies have shown that SNP variation in coding regions, which affects the sequence of amino acids or the folding of peptide chains by changing codons, can alter certain phenotypic traits of organisms [38–40]. However, the effect of regulatory regions on animal phenotypes may be underestimated, and a growing number of studies have found the effects of regulatory regions on phenotypes [41]. In recent studies, regulatory regions have also been found to influence individual phenotypes by regulating gene expression. In humans, ICOS SNPs in 3′ UTR are able to regulate ICOS gene expression by influencing miRNA binding sites [42]. In addition, when the intron region is used as an enhancer, the associated SNP variant affects the expression of genes and may affect obesity in humans [43]. In livestock, variation in the promoter zone may affect the number of litter births in sheep [44], breed differentiation, and fat deposition of pigs [45, 46]. Therefore, as an important regulatory region of gene expression, the promoter region can also change the gene expression level by affecting the binding efficiency of a variety of transcription factors, thereby affecting gene function polymorphisms and animal body development process. So, the study of the variation of the promoter region is of great significance for the future field of animal breeding.

In this study, by querying the functional annotations of all significant genes and identification of transcription factor binding sites in the upstream of gene *CLN8* by ATAC-seq, we identified that *CLN8* is a positive regulator of avian adipocyte differentiation. Moreover, we investigated the effect of these variants on the transcriptional activity of the *CLN8* gene. Our data showed that 5 SNPs found on chromosome 3 are functional polymorphisms and that the mutant haplotype leads to the lower transcriptional activity of the *CLN8* gene than the wild-type, which is consistent with observations from our phenotype-profiling experiments that the wild-type, and heterozygous mutation of haplotype with 5 SNPs significantly decreased the subcutaneous fat weight. We analyzed the transcriptional activity of the upstream regulatory region of the *CLN8* gene through experiments, and further through Alibaba2 website predicting, it was found that there are many adipogenesis-related transcription factors binding sites on its core transcriptional region, including *C/EBPα*, *C/EBPβ*, *C/EBPδ*, *Oct-1*, *Sp1*, *GATA1*, *c-Jun*, *NF-1*, *MYOD*, *AP1*, *Myc,* etc. (Additional file 16), which provide important clues for further exploration of the regulatory mechanism of *CLN8* on avian fat deposition. Further, we found that the mutation can lead to a different binding of transcript factors and affect gene expression in adipocytes. These variant alleles may up-regulate *CLN8* expression through overexpression *C/EBP*α (Additional file 17), which may function as a regulatory factor to combine the upstream gene region to affect expression of *CLN8*. *C/EBP*α is one of the most studied transcription factors in hematopoiesis is the leucine zipper CCAAT-enhancer binding protein, which is mainly involved in cell fate decisions for myeloid differentiation [47]. As a transcription factor, *C/EBP*α can translocate into the nucleus and further regulate a variety of genes directly or indirectly, which are all key factors for cell differentiation [48]. The currently available data are persuasive evidence suggesting that *C/EBP*α expression contributes to the development of fat deposition, and *CLN8* expression as well as its function represents a promising target for gene *C/EBP*α. Therefore, we can further explore the specific mechanism of *C/EBP*α regulating the expression of adipose-promoting factor *CLN8* by combining with candidate causal SNPs through experimental techniques, such as EMSA, ChIP, and their corresponding sequencing analysis techniques.

Previous and current transcriptomic data have shown that a large number of genes are significantly changed during avian adipocyte differentiation [49–51], but their regulatory role in adipocyte differentiation is still poorly understood. In this study, we successfully established an avian *CLN8*^OE^ cell line with the Piggyback technique and discovered that *CLN8* is an important positive regulator of avian adipocyte differentiation. Among these differentially expressed genes, most genes have been reported to have well-defined regulatory functions during adipogenesis in different species. Several potential regulatory pathways could be inferred from the differentially expressed genes and previous studies. For example, *KLF5*, in turn, acts in concert with *C/EBPβ/δ* to activate the *PPARγ2* promoter, and functions as a key component of the transcription factor network controlling adipocyte differentiation [52]. Murine *GATA2* has specifically been expressed in white adipocyte precursors and their down-regulation sets the stage for terminal differentiation. *GATA2* and *GATA3* regulate adipocyte differentiation through molecular control of the preadipocyte-adipocyte transition [53]. In addition to its previously recognized function in suppressing *PPARγ* transcriptional activity, the interaction of *GATA* factors with *C/EBP* is necessary for their ability to negatively regulate adipogenesis [54]. The RNA-Seq results suggest that *CLN8* may positively regulate avian adipogenesis by modulating the expression of a set of genes related to fat cell differentiation, including *PPARG*, which is a core regulator of adipocyte differentiation. Overexpression of the *CLN8* gene could affect these gene expressions directly or indirectly and then effects adipogenesis in avians.

## Conclusions

Avian fat deposition is an important genetic trait that has been related to numerous interesting biological functions, such as energy storage, stress resistance, and immunity. Large-scale genome-wide association analysis identified 35 genes related to duck adipose traits. Among these candidate genes, we demonstrated that the complex transcriptional of *CLN8* is regulated by the 5 linkage SNPs in the upstream, leading to a significantly decreased subcutaneous fat weight in Pekin duck. SNPs in the promoter of *CLN8* can lead to an altered transcriptional activity, and consequently, modulate the adipogenesis in avians. This study presented that promoter mutation of a novel *CLN8* could cause phenotypes changes, and is useful for avian adipose biology and breeding.

## Supplementary Information

**Additional file 1: Table S1.** The primers designed in this study.

**Additional file 2: Table S2.** Basic statistics of Pekin duck phenotypes.

**Additional file 3: Fig. S1.** Population principal component analysis chart.

**Additional file 4: Table S3.** Information for all genome-level significant SNP related to the fat trait.

**Additional file 5: Table S4.** Information for chromosome-level significant SNP related to the fat trait.

**Additional file 6: Fig. S2.** Manhattan and Q-Q plot of association results from genome-wide association analysis of each traits.

**Additional file 7: Table S5.** All SNPs information of the upstream 2K region of *CLN8* gene.

**Additional file 8: Fig. S3.** Typical TSS enrichment plot shows that nucleosome-free fragments are enriched at TSS. The line represents sebum differentiation 0 and 3 d of abdominal fat cells. "F" represents abdominal adipocyte, and "P" represents subcutaneous adipocyte.

**Additional file 9: Fig. S4.** Heatmap of the differentiation of biological replicates of *CLN8* over-expression and control preadipocyte.

**Additional file 10: Table S6.** Statistics relating to the mRNA-seq data.

**Additional file 11: Fig. S5.** The correlation analysis between mRNA-Seq data and RT-PCR results.

**Additional file 12: Table S7.** The list of gene expression profiles related to *CLN8* over-expression and control cells.

**Additional file 13: Table S8.** The RT-PCR result of genes related adipogenesis.

**Additional file 14: Table S9.** The list of DEGs related *CLN8* over-expression and control cells.

**Additional file 15: Table S10.** The GO and KEGG enrichment analysis of DEGs in *CLN8* over-expression and control cells.

**Additional file 16: Table S11.** Prediction of transcription factors in the upstream regulatory region of *CLN8*.

**Additional file 17: Fig. S6.** Overexpression with *C/EBPα* facilitates *CLN8* transcription. (**A**) Transcription factor binding sites analysis of *CLN8* (Wild type -A-T-C-C-G-) ; (**B**) Transcription factor binding sites analysis of *CLN8* (Mutant type -T-C-A-A-C -) ; (**C**  and **D**) The expression of C/EBPα and *CLN8* was detected by RT-qPCR in overexpression- *C/EBPα* transfected and control group. 

## Data Availability

The whole genome sequencing datasets analyzed during the current study are available publicly in the NCBI sequence read archive (PRJNA506902, SRP068685 and PRJNA921894). The RNA-Seq datasets analyzed during the current study are available in the NCBI sequence read archive (PRJNA923472).

## Abbreviations

ATAC-seq: Assay for targeting accessible-chromatin with high-throughout sequencing
ASXL1: ASXL transcriptional regulator 1
ASXL2: ASXL transcriptional regulator 2
ARNTL: Arntl aryl hydrocarbon receptor nuclear translocator-like
ANKRD26: Ankyrin repeat domain containing 26
AP1: Activator protein 1
BMP2: Bone morphogenetic protein 2
BBS12: Bardet-Biedl syndrome 12
CLN8: Ceroid-lipofuscinosis, neuronal 8
C/EBPs: CCAAT-enhancer binding proteins
CCN4: Cellular communication network factor 4
c-Jun: Jun proto-oncogene
AP-1: Transcription factor subunit
ChIP: Chromatin Immunoprecipitation
EMSA: Electrophoretic mobility shift assay
FABP4: Fatty acid binding protein 4
GO: Gene ontology
KEGG: Kyoto Encyclopedia of Genes and Genomes
KLF5: KLF transcription factor 5
LD: Linkage disequilibrium
GATA: GATA binding protein
MAPK14: Mitogen activated protein kinase 14
MYOD: Myogenic differentiation antigen
Myc: Myelocytomatosis
NFR: Nucleosome-free regions
NF-1: Neurofibromin 1
Oct-1: Organic cation/carnitine transporter 1
PC: Principal component
PCR: Polymerase chain reaction
PCA: Principal component analysis
PPARγ: Peroxisome proliferator-activated receptor γ
PTGS2: Prostaglandin-endoperoxide synthase 2
PPARD: Peroxisome proliferator activated receptor delta
PPARG: Peroxisome proliferator activated receptor gamma
QTL: Quantitative trait locus
Q-Q plot: Quantile–Quantile Plot
RT-qPCR: Real-time fluorescence quantification PCR
RNA-Seq: High-throughput sequencing of RNA
RUNX1T1: RUNX1 partner transcriptional co-repressor 1
SNP: Single nucleotide polymorphism
STK4: Serine/threonine kinase 4
Sp1: Sp1 transcription factor
TSS: Transcription start site
YAP1: Yes associated transcriptional regulator 1
ZC3H12A: Zinc finger CCCH-type containing 12A
ZFPM1: Zinc finger protein, FOG family member 1

## References

[1] Ding S, Li G, Chen S, Zhu F, Hao J, Yang F (2021). Comparison of carcass and meat quality traits between lean and fat Pekin ducks. Anim Biosci.

[2] Zhu F, Yin Z, Wang Z, Smith J, Zhang F, Martin F, et al. Three chromosome-level duck genome assemblies provide insights into genomic variation during domestication. Nat Commun. 2021;12:5932. 10.1038/s41467-021-26272-1. 10.1038/s41467-021-26272-1 PMC8505442 34635656

[3] Jo J, Ga O, Pack S, Jou W, Mullen S, Sumner AE, et al. Hypertrophy and/or hyperplasia: Dynamics of adipose tissue growth. PLoS Comput Biol. 2009;5(3):e1000324. 10.1371/journal.pcbi.1000324. 10.1371/journal.pcbi.1000324 PMC2653640 19325873

[4] Wang QA, Tao C, Gupta RK, Scherer PE (2013). Tracking adipogenesis during white adipose tissue development, expansion and regeneration. Nat Med.

[5] Abd BA, Chen J, Nie Q, Zhang X (2018). Genomic insights into the multiple factors controlling abdominal fat deposition in a chicken model. Front Genet.

[6] Lee J, Schmidt H, Lai B, Ge K. Transcriptional and epigenomic regulation of adipogenesis. Mol Cell Biol. 2019;39(11). 10.1128/MCB.00601-18. 10.1128/MCB.00601-18 PMC6517598 30936246

[7] Wang Z, Yin Z, Zhang F, Li X, Chen S, Yang N, et al. Dynamics of transcriptome changes during subcutaneous preadipocyte differentiation in ducks. BMC Genom.2019;20:688. 10.1186/s12864-019-6055-9. 10.1186/s12864-019-6055-9 PMC6720933 31477016

[8] Wang Z, Zhao Q, Li X, Yin Z, Chen S, Wu S, et al. MYOD1 inhibits avian adipocyte differentiation via miRNA-206/KLF4 axis. J Anim Sci Biotechnol. 2021;12:55. 10.1186/s40104-021-00579-x. 10.1186/s40104-021-00579-x PMC8101123 33952351

[9] Sun D, Li X, Yin Z, Hou Z. The Full-Length transcriptome provides new insights into the transcript complexity of abdominal adipose and subcutaneous adipose in pekin ducks. Front Physiol. 2021;12:767739. 10.3389/fphys.2021.767739. 10.3389/fphys.2021.767739 PMC8631521 34858212

[10] Hu Z, Park CA, Reecy JM (2022). Bringing the Animal QTLdb and CorrDB into the future: Meeting new challenges and providing updated services. Nucleic Acids Res.

[11] Hu Z, Park CA, Reecy JM (2016). Developmental progress and current status of the Animal QTLdb. Nucleic Acids Res.

[12] Zhou Z, Li M, Cheng H, Fan W, Yuan Z, Gao Q, et al. An intercross population study reveals genes associated with body size and plumage color in ducks. Nat Commun. 2018;9:2648. 10.1038/s41467-018-04868-4. 10.1038/s41467-018-04868-4 PMC6050300 30018292

[13] Xu Y, Liu H, Jiang Y, Fan W, Hu J, Zhang Y (2019). Genome-wide association studies reveal genetic loci associated with plasma cholinesterase activity in ducks. Anim Genet.

[14] Zhu F, Cui Q, Hou Z. SNP discovery and genotyping using Genotyping-by-Sequencing in Pekin ducks. Sci Rep. 2016;6:36223. 10.1038/srep36223. 10.1038/srep36223 PMC5109183 27845353

[15] Deng MT, Zhu F, Yang YZ, Yang FX, Hao JP, Chen SR (2019). Genome-wide association study reveals novel loci associated with body size and carcass yields in Pekin ducks. BMC Genom.

[16] Zhu F, Cheng S, Yang Y, Hao J, Yang F, Hou Z (2019). Genome-Wide association study of growth and feeding traits in pekin ducks. Front Genet.

[17] Deng MT, Zhang F, Zhu F, Yang YZ, Yang FX, Hao JP (2020). Genome-wide association study reveals novel loci associated with fat-deposition and meat-quality traits in Pekin ducks. Anim Genet.

[18] Lin F, Zhu F, Hao J, Yang F, Hou Z (2018). In vivo prediction of the carcass fatness using live body measurements in Pekin ducks. Poult Sci.

[19] Li H, Durbin R (2009). Fast and accurate short read alignment with Burrows-Wheeler transform. Bioinformatics.

[20] McKenna A, Hanna M, Banks E, Sivachenko A, Cibulskis K, Kernytsky A (2010). The Genome Analysis Toolkit: A MapReduce framework for analyzing next-generation DNA sequencing data. Genome Res.

[21] Browning BL, Browning SR (2016). Genotype imputation with millions of reference samples. Am J Hum Genet.

[22] McLaren W, Gil L, Hunt SE, Riat HS, Ritchie GRS, Thormann A, et al. The ensembl variant effect predictor. Genome Biol. 2016;17:122. 10.1186/s13059-016-0974-4. 10.1186/s13059-016-0974-4 PMC4893825 27268795

[23] Zhou X, Stephens M (2012). Genome-wide efficient mixed-model analysis for association studies. Nat Genet.

[24] Turley P, Walters RK, Maghzian O, Okbay A, Lee JJ, Fontana MA (2018). Multi-trait analysis of genome-wide association summary statistics using MTAG. Nat Genet.

[25] Heinz S, Benner C, Spann N, Bertolino E, Lin YC, Laslo P (2010). Simple combinations of Lineage-Determining transcription factors prime cis-Regulatory elements required for macrophage and b cell identities. Mol Cell.

[26] Li H, Handsaker B, Wysoker A, Fennell T, Ruan J, Homer N (2009). The Sequence Alignment/Map format and SAMtools. Bioinformatics.

[27] Zhang Y, Liu T, Meyer CA, Eeckhoute J, Johnson DS, Bernstein BE (2008). Model-based analysis of ChIP-Seq (MACS). Genome Biol.

[28] Ramírez F, Dündar F, Diehl S, Grüning BA, Manke T (2014). DeepTools: A flexible platform for exploring deep-sequencing data. Nucleic Acids Res.

[29] Love MI, Huber W, Anders S. Moderated estimation of fold change and dispersion for RNA-seq data with DESeq2. Genome Biol. 2014;15:550. 10.1186/s13059-014-0550-8. 10.1186/s13059-014-0550-8 PMC4302049 25516281

[30] Wang W, Zhang T, Wu C, Wang S, Wang Y, Li H, et al. Immortalization of chicken preadipocytes by retroviral transduction of chicken TERT and TR. PLoS One. 2017;12(5):e177348. 10.1371/journal.pone.0177348. 10.1371/journal.pone.0177348 PMC5423695 28486516

[31] Shang Z, Guo L, Wang N, Shi H, Wang Y, Li H (2014). Oleate promotes differentiation of chicken primary preadipocytesin vitro. Biosci Rep..

[32] Livak KJ, Schmittgen TD (2001). Analysis of relative gene expression data using Real-Time quantitative PCR and the 2−ΔΔCT method. Methods.

[33] Bolger AM, Lohse M, Usadel B (2014). Trimmomatic: A flexible trimmer for Illumina sequence data. Bioinformatics.

[34] Patro R, Duggal G, Love MI, Irizarry RA, Kingsford C (2017). Salmon provides fast and bias-aware quantification of transcript expression. Nat Methods.

[35] Grabe N (2002). AliBaba2: Context specific identification of transcription factor binding sites. In Silico Biol.

[36] Zhou Y, Zhou B, Pache L, Chang M, Khodabakhshi AH, Tanaseichuk O, et al. Metascape provides a biologist-oriented resource for the analysis of systems-level datasets. Nat Commun. 2019;10:1523. 10.1038/s41467-019-09234-6. 10.1038/s41467-019-09234-6 PMC6447622 30944313

[37] Castro-Mondragon JA, Riudavets-Puig R, Rauluseviciute I, Berhanu Lemma R, Turchi L, Blanc-Mathieu R (2022). JASPAR 2022: The 9th release of the open-access database of transcription factor binding profiles. Nucleic Acids Res.

[38] Kimchi-Sarfaty C, Oh JM, Kim I, Sauna ZE, Calcagno AM, Ambudkar SV (2007). A "silent" polymorphism in the MDR1 gene changes substrate specificity. Science.

[39] Jones M, Sergeant C, Richardson M, Groth D, Brooks S, Munyard K (2019). A non-synonymous SNP in exon 3 of the KIT gene is responsible for the classic grey phenotype in alpacas (Vicugna pacos). Anim Genet.

[40] Matsumoto H, Kohara R, Sugi M, Usui A, Oyama K, Mannen H, et al. The non-synonymous mutation in bovine SPP1 gene influences carcass weight. Heliyon. 2019;5(12):e3006. 10.1016/j.heliyon.2019.e03006. 10.1016/j.heliyon.2019.e03006 PMC6920195 31879711

[41] Klees S, Heinrich F, Schmitt A, Gültas M (2021). AgReg-SNPdb: A database of regulatory SNPs for agricultural animal species. Biology.

[42] Conteduca G, Rossi A, Megiorni F, Parodi A, Ferrera F, Tardito S, et al. Single‐nucleotide polymorphisms in 3′‐untranslated region inducible costimulator gene and the important roles of miRNA in alopecia areata. Skin Health Dis. 2021;1:e34. 10.1002/ski2.34. 10.1002/ski2.34 PMC9060044 35664973

[43] Dong SS, Zhu DL, Zhou XR, Rong Y, Zeng M, Chen JB (2021). An intronic risk SNP rs12454712 for central obesity acts as an Allele-Specific enhancer to regulate BCL2 expression. Diabetes.

[44] Sahoo SS, Mishra C, Kaushik R, Rout PK, Singh MK, Bhusan S (2021). Association of a SNP in KISS 1 gene with reproductive traits in goats. Biol rhythm res.

[45] Wu Q, Yu H, Wei W, Cheng Y, Huang S, Shi H (2018). Linkage disequilibrium and functional analysis of PRE1 insertion together with SNPs in the promoter region of IGFBP7 gene in different pig breeds. J Appl Genet.

[46] Wang L, Chao Z, Wang Y (2018). Identification of two novel single nucleotide polymorphisms in the promoter region of the pig AMP deaminase 1 gene associated with carcass traits. DNA Cell Biol.

[47] Avellino R, Delwel R (2017). Expression and regulation of C/EBPa in normal myelopoiesis and in malignant transformation. Blood.

[48] Song G, Wang L, Bi K, Jiang G (2015). Regulation of the C/EBPα signaling pathway in acute myeloid leukemia (Review). Oncol Rep.

[49] Liu TM, Martina M, Hutmacher DW, Hui JHP, Lee EH, Lim B (2007). Identification of common pathways mediating differentiation of bone marrow- and adipose Tissue-Derived human mesenchymal stem cells into three mesenchymal lineages. Stem Cells.

[50] Cristancho AG, Lazar MA (2011). Forming functional fat: A growing understanding of adipocyte differentiation. Nat Rev Mol Cell Biol.

[51] Cawthorn WP, Scheller EL, MacDougald OA (2012). Adipose tissue stem cells meet preadipocyte commitment: Going back to the future. J Lipid Res.

[52] Oishi Y, Manabe I, Tobe K, Tsushima K, Shindo T, Fujiu K (2005). Krüppel-like transcription factor KLF5 is a key regulator of adipocyte differentiation. Cell Metab.

[53] Tong Q, Dalgin G, Xu H, Ting CN, Leiden JM (2000). Function of GATA transcription factors in preadipocyte-adipocyte transition. Sci.

[54] Tong Q, Tsai J, Tan G, Dalgin G, Hotamisligil GS (2005). Interaction between GATA and the C/EBP family of transcription factors is critical in GATA-Mediated suppression of adipocyte differentiation. Mol Cell Biol.

