# Supplementary material for: A novel candidate gene CLN8 regulates fat deposition in avian
Source: J Anim Sci Biotechnol. 2023 May 1;14:70. doi: 10.1186/s40104-023-00864-x (PMC10150489; doi:10.1186/s40104-023-00864-x)
Supplement: Supplementary file 2 — Additional file 2: Table S2. Basic statistics of Pekin duck phenotypes. [file 40104_2023_864_MOESM2_ESM.docx]

**Table S2** Basic statistics of Pekin duck phenotypes

|  | **Pop2014** | | **Pop2019** | | **Pop2020** | |
| --- | --- | --- | --- | --- | --- | --- |
| **Trait** | **Count** | **Average ± SD** | **Count** | **Average ± SD** | **Count** | **Average ± SD** |
| BW42, g | 632 | 3072.91 ± 245.53^a^ | 624 | 3332.75±253.40^c^ | 618 | 3393.41 ± 248.15^c^ |
| SFW, g | 632 | 708.00 ± 90.04^a^ | 624 | 828.15±92.51^c^ | 624 | 970.18 ± 114.47^c^ |
| SFP, % | 632 | 32.73 ± 2.87^a^ | 622 | 35.01±2.52^c^ | 623 | 38.11 ± 2.46^c^ |
| AFW, g | 632 | 52.68 ± 13.64^a^ | 621 | 69.01±14.63^c^ | 624 | 88.29 ± 16.75^c^ |
| AFP, % | 632 | 2.33 ± 0.55^a^ | 623 | 2.85±0.56^c^ | 623 | 3.35 ± 0.56^c^ |

Abbreviations: BW42, body weight at day 42; SFW: skin fat weight; SFP: skin fat percentage; AFW: abdominal fat weight; AFP: abdominal fat percentage

^a,c^Different superscripts in the same row indicate significant differences (*P* < 0.01)
