# Supplementary figures and images for: A novel candidate gene CLN8 regulates fat deposition in avian
Source: J Anim Sci Biotechnol. 2023 May 1;14:70. doi: 10.1186/s40104-023-00864-x (PMC10150489; doi:10.1186/s40104-023-00864-x)

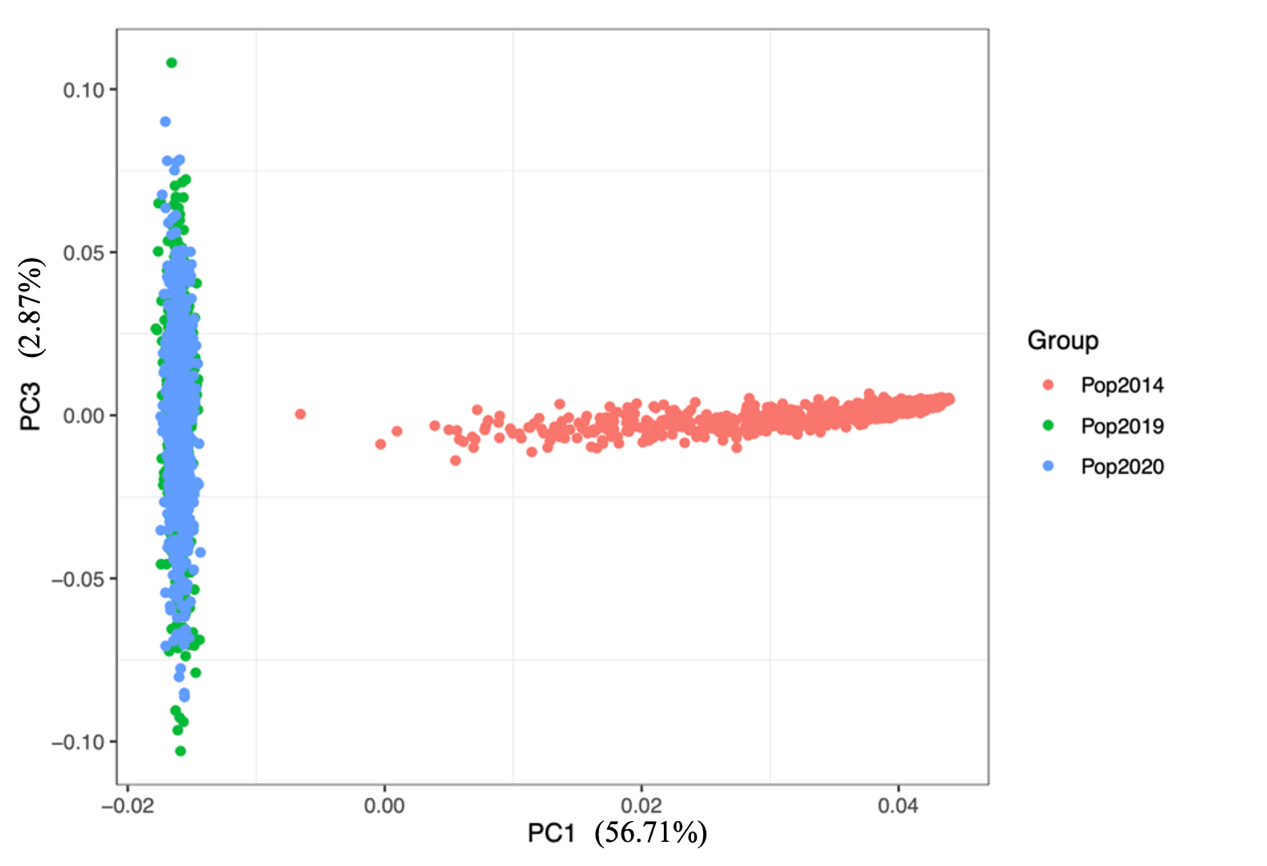

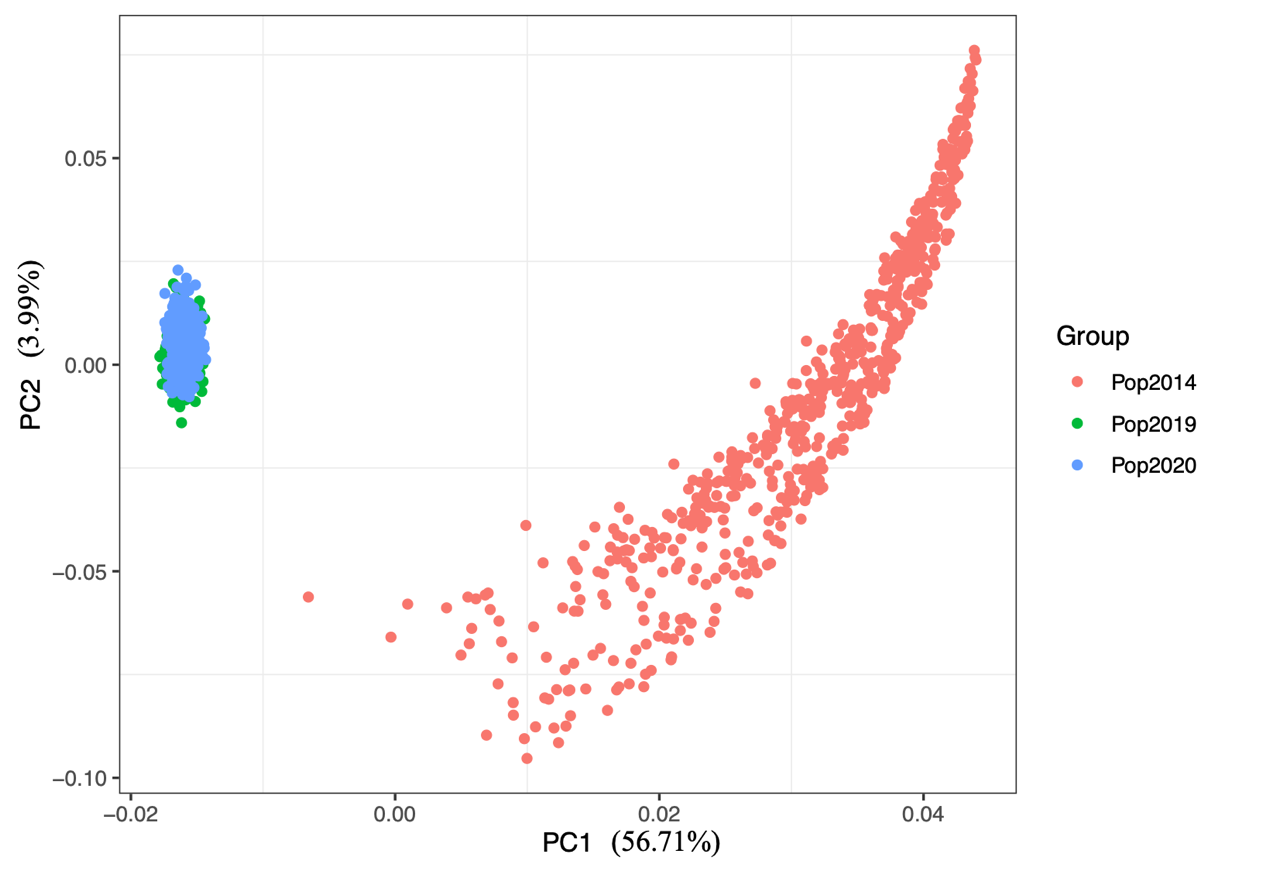


**Fig. S1**Population principal component analysis chart

Supplement: Supplementary file 3 — Additional file 3: Fig. S1. Population principal component analysis chart. [file 40104_2023_864_MOESM3_ESM.docx]

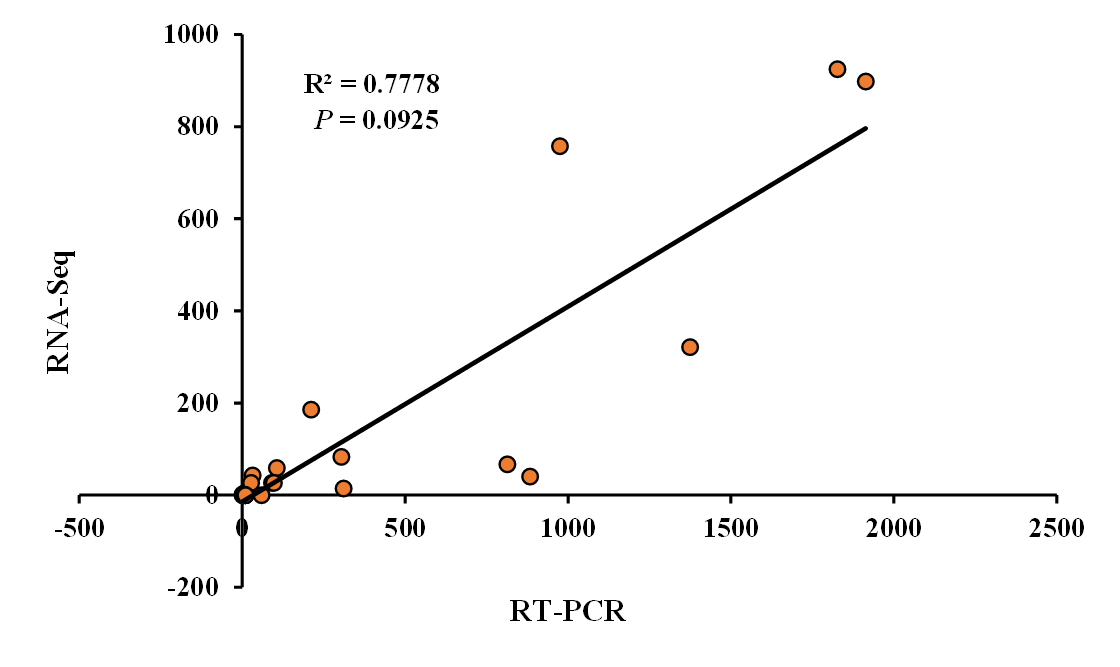


**Fig. S5** The correlation analysis between RNA-Seq data and RT-PCR results

Supplement: Supplementary file 11 — Additional file 11: Fig. S5. The correlation analysis between mRNA-Seq data and RT-PCR results. [file 40104_2023_864_MOESM11_ESM.docx]
