# Supplementary material for: A novel candidate gene CLN8 regulates fat deposition in avian
Source: J Anim Sci Biotechnol. 2023 May 1;14:70. doi: 10.1186/s40104-023-00864-x (PMC10150489; doi:10.1186/s40104-023-00864-x)

**Fig. S2.** Manhattan and Q-Q plot of association results from genome-wide association analysis of each traits.


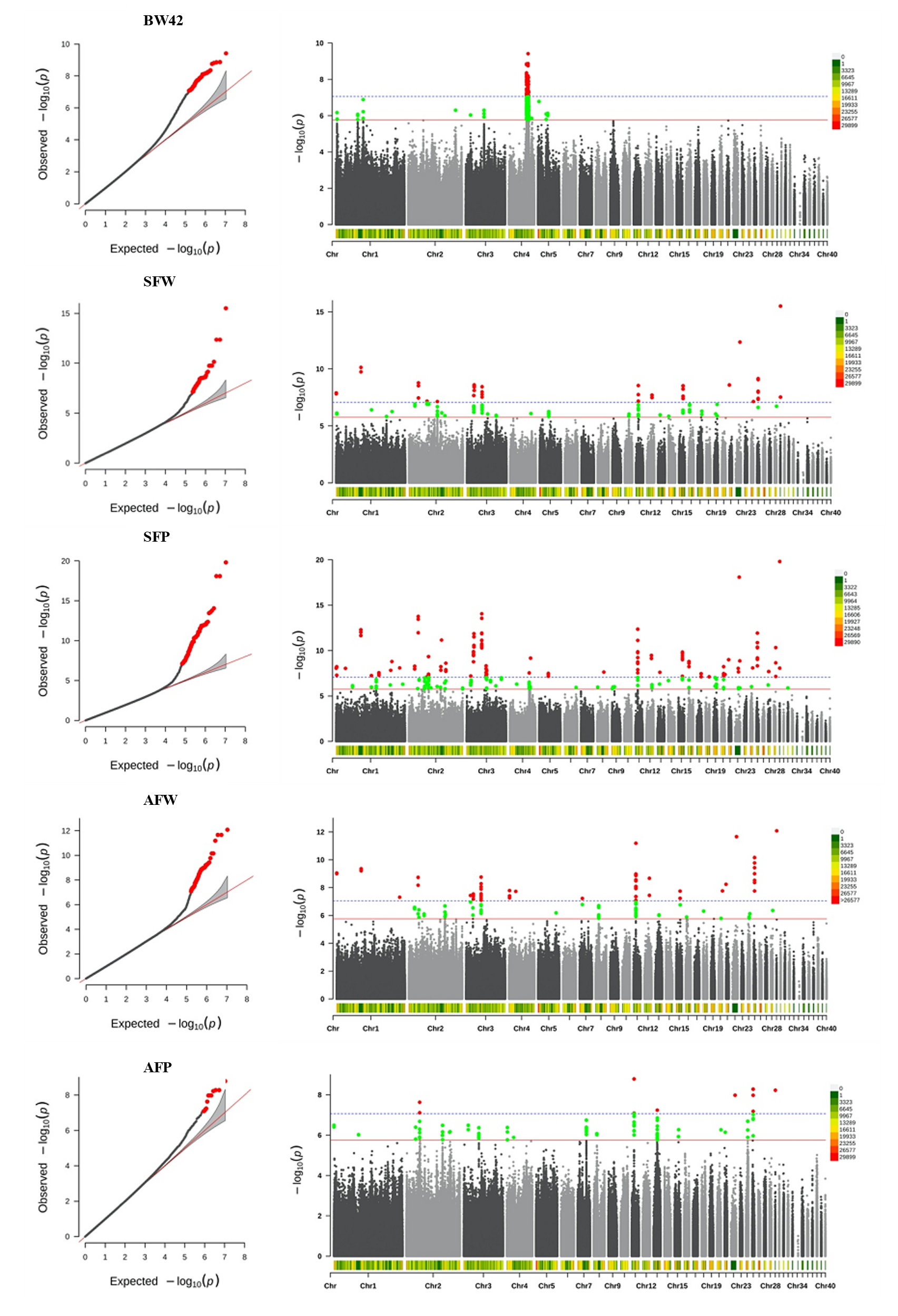

Supplement: Supplementary file 6 — Additional file 6: Fig. S2. Manhattan and Q-Q plot of association results from genome-wide association analysis of each traits. [file 40104_2023_864_MOESM6_ESM.docx]
