# Supplementary material for: A novel candidate gene CLN8 regulates fat deposition in avian
Source: J Anim Sci Biotechnol. 2023 May 1;14:70. doi: 10.1186/s40104-023-00864-x (PMC10150489; doi:10.1186/s40104-023-00864-x)
Supplement: Supplementary file 8 — Additional file 8: Fig. S3. Typical TSS enrichment plot shows that nucleosome-free fragments are enriched at TSS. The line represents sebum differentiation 0 and 3 d of abdominal fat cells. "F" represents abdominal adipocyte, and "P" represents subcutaneous adipocyte. [file 40104_2023_864_MOESM8_ESM.docx]

**
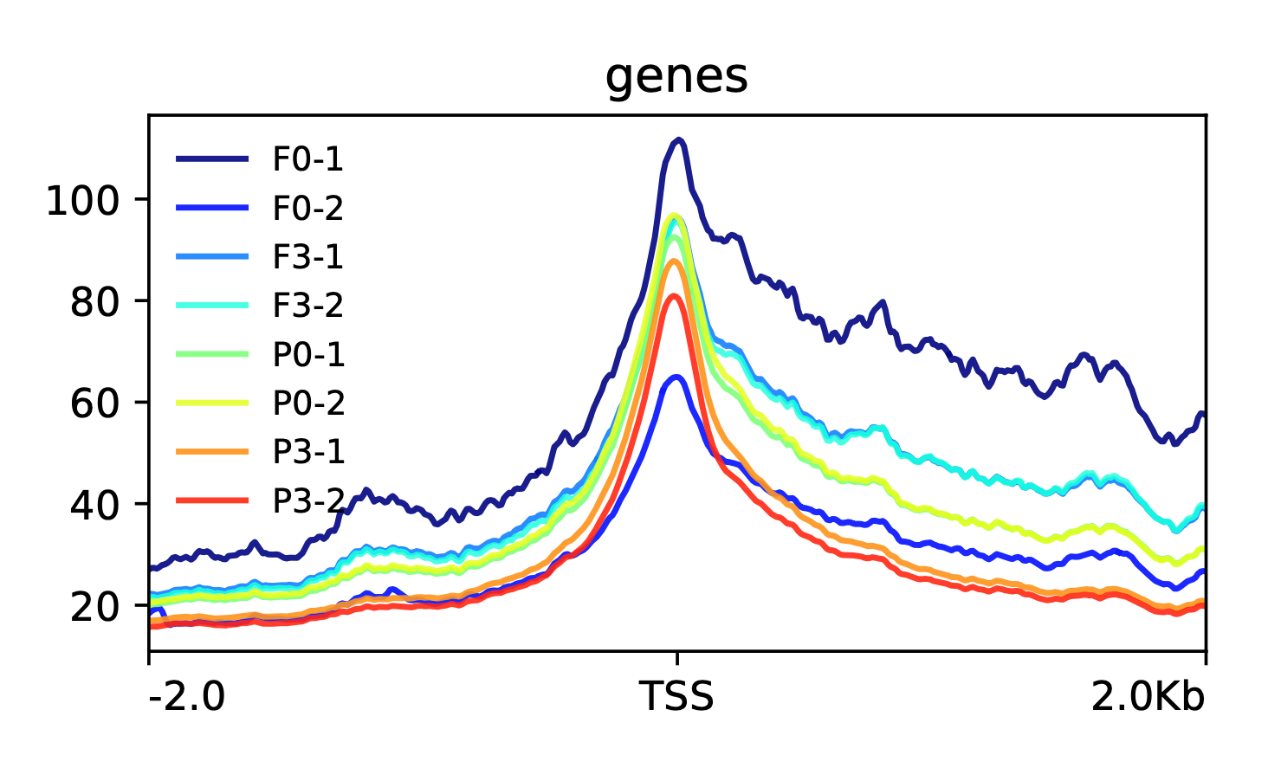
**

**Fig. S3** Typical TSS enrichment plot shows that nucleosome-free fragments are enriched at TSS. The line represents differentiation 0 and 3 d of adipocyte. "F" represents abdominal adipocyte, and "P" represents subcutaneous adipocyte
