# Supplementary material for: A novel candidate gene CLN8 regulates fat deposition in avian
Source: J Anim Sci Biotechnol. 2023 May 1;14:70. doi: 10.1186/s40104-023-00864-x (PMC10150489; doi:10.1186/s40104-023-00864-x)
Supplement: Supplementary file 9 — Additional file 9: Fig. S4. Heatmap of the differentiation of biological replicates of CLN8 over-expression and control preadipocyte. [file 40104_2023_864_MOESM9_ESM.docx]

**Fig. S4** Heatmap of the differentiation of biological replicates of preadipocyte. The colors ranging from white to blue represent Pearson correlation coefficients ranging from 0 to 1, indicating low to high correlations, respectively
