# Supplementary material for: A novel candidate gene CLN8 regulates fat deposition in avian
Source: J Anim Sci Biotechnol. 2023 May 1;14:70. doi: 10.1186/s40104-023-00864-x (PMC10150489; doi:10.1186/s40104-023-00864-x)
Supplement: Supplementary file 17 — Additional file 17: Fig. S6. Overexpression with C/EBPα facilitates CLN8 transcription. (A) Transcription factor binding sites analysis of CLN8 (Wild type -A-T-C-C-G-) ; (B) Transcription factor binding sites analysis of CLN8 (Mutant type -T-C-A-A-C -) ; (C and D) The expression of C/EBPα and CLN8 was detected by RT-qPCR in overexpression- C/EBPα transfected and control group. [file 40104_2023_864_MOESM17_ESM.docx]

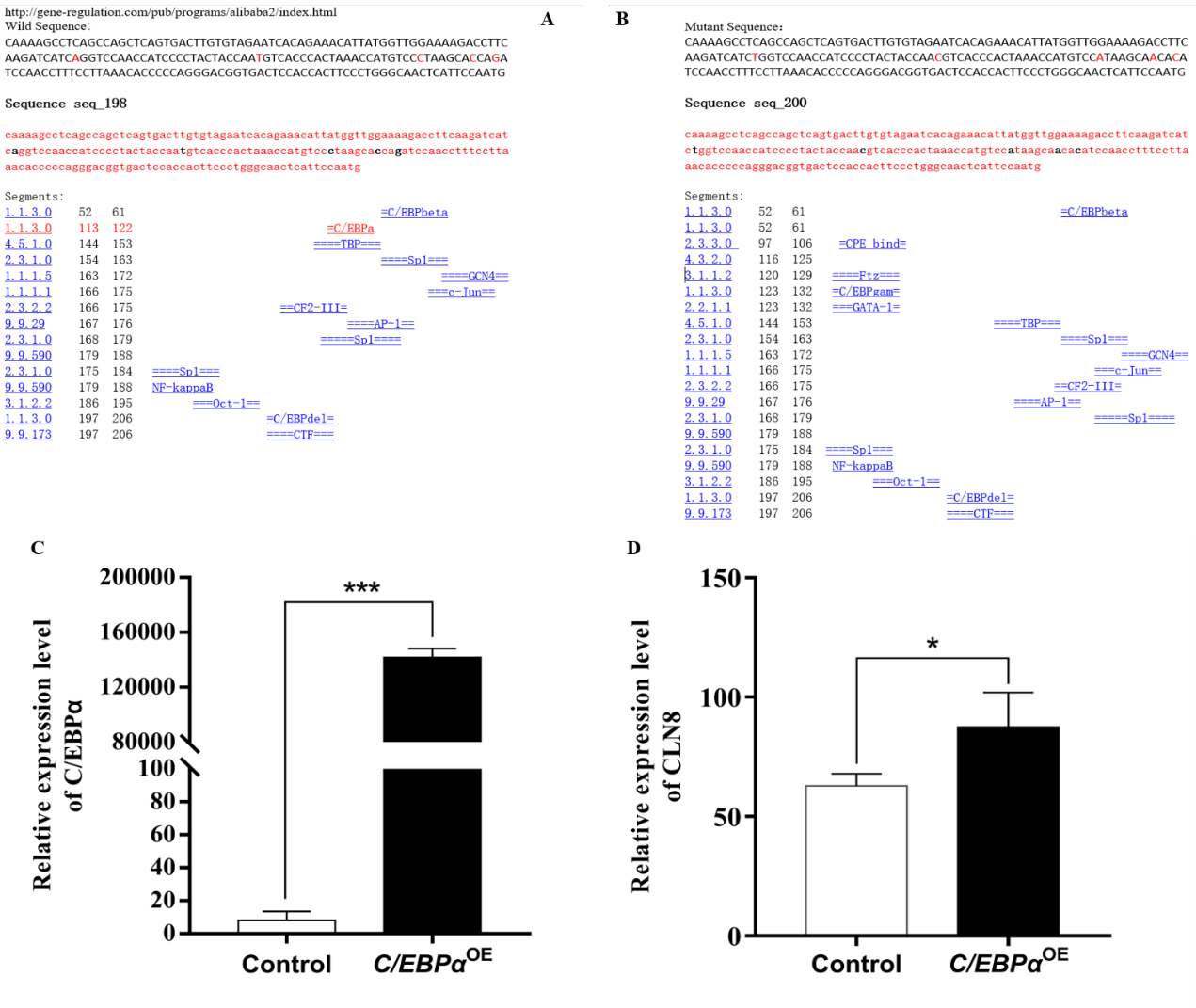


**Fig. S6** Overexpression with *C/EBPα* facilitates *CLN8* transcription. (**A**) Transcription factor binding sites analysis of CLN8 (Wild type -A-T-C-C-G-) ; (**B**) Transcription factor binding sites analysis of CLN8 (Mutant type -T-C-A-A-C -) ; (**C** and **D**) The expression of *C/EBPα* and *CLN8* was detected by qRT-PCR in overexpression- *C/EBPα* transfected and control group
